# Virus Infection Recognition and Early Innate Responses to Non-Enveloped Viral Vectors

**DOI:** 10.3390/v2010244

**Published:** 2010-01-19

**Authors:** Dmitry M. Shayakhmetov

**Affiliations:** Division of Medical Genetics, Department of Medicine, University of Washington, Seattle, WA 98195-7720, USA; E-Mail: dshax@u.washington.edu; Tel.: +1-206-543-6773; Fax: +1-206-685-8675

**Keywords:** virus vectors, innate immunity, inflammation

## Abstract

Numerous human genetic and acquired diseases could be corrected or ameliorated if viruses are harnessed to safely and effectively deliver therapeutic genes to diseased cells and tissues *in vivo*. Innate immune and inflammatory response represents one of the key stumbling blocks during the development of viral-based therapies. In this review, current data on the early innate immune responses to viruses and to the most commonly used gene therapy vectors (using adenovirus and adeno-associated virus) will be discussed. Recent findings in the field may help develop new approaches to moderate these innate immune anti-viral responses and thus improve the safety of viral vectors for human gene therapy applications.

## 1. Introduction

**Figure 1.**
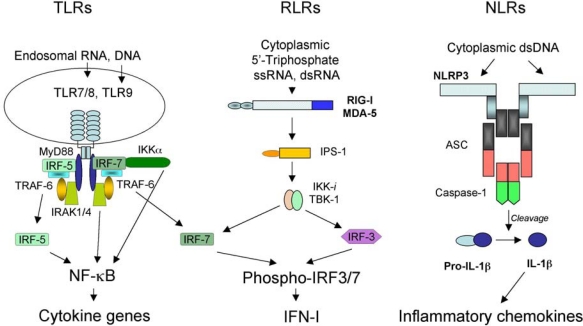
Three classes of pattern recognition receptors engaged in sensing viral nucleic acids in infected cells. See text for the detail description of their respective structures, ligand specificities, and roles in inducing host antiviral responses.

During the millions of years of co-evolution, viral pathogens and their hosts evolved to an equilibrium allowing both hosts and viruses to coexist and thus survive. However, this equilibrium is quasi-stationary due to constant adaptation of viruses to existing or new hosts and to eternal selection within these hosts for the most effective antiviral mechanisms. One of the best testaments to the quasi-stationary nature of the balance between viruses and their hosts is a set of recent reports of unanticipated severe innate immune and inflammatory responses to rather benign viral pathogens used as gene delivery vehicles in human gene therapy trials [1–3]. Viruses are obligate pathogens and are very efficient at infecting host cells that support their reproduction and dissemination. Clinical studies suggest that, in many cases, a small number of virus particles is sufficient to transmit virus-associated disease [4]. Moreover, viruses develop an array of factors and strategies that allow them to evade or modulate the host immune system. These factors facilitate rapid completion of the viral reproductive cycle, contributing to the spread of infection. In response to the ever-present threat from viruses, hosts have evolved molecular mechanisms to detect virus infection and limit virus-induced damage. Abundant information has recently emerged on the molecular mechanism of innate immunity to virus infection in mammals [5]. One of the fundamental principles of virus recognition by the host innate immune system appears to rely on the sensing of virus-associated nucleic acids in infected cells by specialized classes of receptors – Toll-like receptors (TLRs), retinoid acid-inducible gene I (RIG-I)-like receptors (RLRs), and nucleotide oligomerization domain (NOD)-like receptors (NLRs) (Figure 1). This latest evidence also indicates that the host recognition of cell damage or stress induced merely by virus entry into cells is likely to be the second key principle for virus sensing which does not rely on pathogen-associated nucleic acid recognition.

## 2. Molecular Basis for Cellular Recognition of Virus Infection

The obligate nature of viral pathogens creates a considerable challenge for the host to detect onset and continuation of virus infection. Because viruses replicate in host cells and frequently utilize host protein and nucleic acid processing machineries, host/non-host discrimination becomes complicated based solely on pathogen-specific chemical moiety recognition. However, the natural diversity of virus-associated nucleic acids makes them a legitimate target for detection within infected cells. While mammalian cells under normal physiologic conditions possess only a limited number of nucleic acid types, such as double-stranded (ds) DNA with methylated CpG motifs and single-stranded (ss) RNA with a cap structure at the 5′ end of the molecule, the structure of virus-associated nucleic acids is very diverse. Viruses encode their genetic information in a form of ssDNA, linear dsDNA, circular dsDNA, ssRNA, dsRNA, all with myriads of unique modifications and permutations of polarity and replicative intermediates. Accumulating evidence suggests that host recognition of virus-associated nucleic acids is a fundamental principle for sensing virus infection in mammalian cells.

### 2.1. TLR-dependent recognition of virus infection

One of the best-studied families of receptors mediating pathogen recognition by the innate immune system are TLRs. There are more than 10 distinct TLR receptors identified in mammals to date [6]. TLR3, TLR7, and TLR9 were shown to recognize viral-associated nucleic acids in the cellular endosomal compartment. TLR3 is activated by a dsRNA [7], TLR7 is activated by a ssRNA [8–10], and TLR9 is activated by an unmethylated CpG DNA motif [11]. While TLR3 is expressed in many cell types, TLR7 and TLR9 are expressed at high levels in plasmacytoid dendritic cells (pDCs) [12]. All TLR-initiated signaling converges on the activation of type I interferon (IFN-I) through the engagement of IRF3 and/or IRF7 transcription factors and the early response inflammatory cytokine genes via activation of NF-κB [13] (Figure 1). Despite this convergence on a specific set of genes, the signaling pathways that lead to IFN-I and cytokine gene activation in response to TLR engagement are cell-type specific and recruit different adaptors and mediators depending on the type of TLR. dsRNA binding to TLR3 ecto-domain in the endosomes leads to receptor dimerization and the recruitment of an adaptor molecule TRIF (also known as TICAM-1) [14,16]. TRIF then binds TRAF3 and TRAF6 proteins via TRAF-binding motifs that are present within N-terminal region of TRIF [17]. TRAF6 is responsible for activating NF-κB that leads to the expression of pro-inflammatory cytokines [18]. TRIF, however, can also activate TBK1 and IKKi protein kinases, which phosphorylate IRF3 and lead to its translocation into the nucleus and activation of IFN-I [19,20]. TLR3 plays critical role in controlling replication of MCMV in mice as well as purified reovirus genomic dsRNA is a potent activator of IFN-I in a TLR3-dependent manner [7,21].

In the cellular endosomal compartment, TLR7 and TLR9 recognize ssRNA and unmethylated CpG DNA, respectively [8–11]. Upon engagement of a cognate ligand, TLR7 and TLR9 initiate signaling through a common adaptor molecule myeloid differentiation factor 88 (MyD88) [22]. MyD88 then interacts with IL-1R-associated kinase 4 (IRAK-4) [5]. IRAK-4, in turn, transduces the signal though IRAK-1 and IRAK-2, that leads to the activation of TRAF6. Activation of TRAF6, through a set of protein kinases, results in phosphorylation of mitogen activated protein kinase 6 (MAPK6) and IKK-β, which modulate the activation of NF-κB and MAPKs leading to the production of pro-inflammatory cytokines. In pDCs, activation of TLR7 and TLR9, in addition to inflammatory cytokine production, leads to a MyD88-dependent activation of IRF7 which is responsible for the induction of IFN-I [23,24]. In pDCs, vesicular stomatitis virus (VSV) RNA is recognized by TLR7 upon autophagosome formation [25]. In response to adenovirus infection, pDCs *in vitro* activate IFN-I production in a TLR-9-dependent manner [26].

### 2.2. RLR-dependent recognition of virus infection

Upon entry into host cells, many viral pathogens (specifically those with lipid envelopes such as influenza and human immunodeficiency virus [HIV]) avoid exposure of their genomic nucleic acids to endosomal TLRs. However, activation of IFN-I in response to virus infection is afforded through a cytoplasmic detection of viral RNAs by an RLR family of receptors, consisting of RIG-I and melanoma differentiation-associated gene 5 (MDA5) [27–29]. RIG-I and MDA5 directly bind viral RNA via the helicase domain [30] (Figure 1). Although both RIG-I and MDA-5 can bind dsRNA, MDA-5 activates IFN-I production upon binding of long (>2kb, dsRNA species [31]) while RIG-I induces IFN-I upon binding of short dsRNA and 5′-triphosphate-containing ssRNA [32–34]. Similarly to the TLR receptor family, IFN-I and inflammatory cytokine genes are the principal targets of RLR receptors’ signaling. When the viral RNA binds to and activates RIG-I or MDA5, these proteins engage key adapter protein IFN-β promoter stimulator-1 (IPS-1), also known as mitochondrial antiviral (MAVS), virus-induced signaling adapter (VISA), or CARD adapter inducing IFN-β (CARDIFF) [35–38]. Activated IPS-1 recruits TNFR-associated death domain protein (TRADD) which forms a complex with TRAF3 and receptor-interacting protein RIP-1 [39]. TRAF3 is critical for IFN-I induction and mediates activation of TBK1 and IKKi kinases which phosphorylate IRF3 and IRF7, leading to activation of IFN-I and a set of IFN-I-inducible genes [20,40]. IPS-1 also is critical for the activation of inflammatory cytokine genes mediated by NF-κB via phosphorylation of classical IKKα/β kinases.

MDA-5 and RIG-I are important for activating host responses to RNA viruses. RIG-I was shown to activate IFN-I in response to paramyxoviruses, VSV, influenza virus, hepatitis C virus, and Japanese encephalitis virus infection [41–44]. MDA5 is critical for IFN-I production in response to reoviruses [31] and picornaviruses, including encephalomyocarditis virus (ECMV) and Theiler’s virus [45]. Mice that lack both MDA5 and RIG-I are highly susceptible to VSV and EMCV virus infection [5,29]. Collectively, cytoplasmic detection of viral RNAs by RLR appears to be the key pathway of host innate immunity activation by RNA viral pathogens.

### 2.3. NLR-dependent recognition of virus infection

NLR family consists of a relatively large number of intracellular receptors with a prototypic tripartite structure [46,47]. The N-terminus is composed of either a caspase recruitment domain (CARD) or a Pyrin domain (PYD) that are important for signal transduction. The central part of the NLR molecule is composed of a nucleotide-binding domain (NBD) critical for ATP binding and oligomerization. The C-terminus is composed of a leucine-rich repeat (LRR) domain, important for ligand binding and autoregulation of the NLR function [48]. Upon engagement of a LRR-specific ligand, NBD binds ATP, leading to oligomerization of the NLR and initiation of a signal transduction via N-terminal domain binding specific adaptors, then leads to the activation of MAPK kinases and NF-κB (in case of a PYD N-terminal domain), or association of NLR with a supramolecular complex of proteins called “inflammasome” via CARD domain [49]. In addition to an NLR, inflammasome includes ASC adapter protein and inflammatory caspases, such as caspase-1 [50] (Figure 1). Upon activation of NLR and its recruitment into the inflammasome complex, pro-caspase-1 is processed into functionally active caspase-1, which further cleaves pre-IL-1β into mature IL-1β, leading to its release from cells and activation of the IL-1R signaling pathway.

There is abundant evidence demonstrating the essential role of NLRs in sensing and controlling microbial infection in mammalian cells [51]. Regarding the NLR involvement in recognition of viral infection, recent data indicate that the NLR family member NLRP3 (also called Cryopyrin) plays an essential role in sensing viral and microbial DNA in macrophages *in vitro* [52]. Recent data also suggest that NLRP3 mediates recognition of influenza A virus infection [53,54]. Mice deficient for NLRP3 exhibit dramatically increased mortality and reduced immune response to the influenza virus. NLRP3 inflammasome activation by influenza virus was dependent on lysosomal maturation and reactive oxygen species. It was also suggested that NLRP3 inflammasome is involved in sensing viral RNA [53], while another study also implicates NLRP3 inflammasome in the resolution of inflammation [54].

### 2.4. Recognition of cytoplasmic dsDNA by DAI and AIM2

Intra-cytoplasmic detection of dsDNA represents an important mechanism for the detection of viral and microbial pathogens. Although several studies clearly showed that cells transfected with dsDNA leads to activation of IFN-I and NF-κB-dependent inflammatory cytokines, the molecular sensors of dsDNA in the cytoplasm that remained unidentified until recently. Takaoka *et al*. showed that dsDNA-dependent activation of IFN-I production in cells is mediated by a protein named DNA-dependent activator of IFN-regulatory factors (DAI) [55]. DAI was also known as Z-DNA binding protein ZBP-1 and DLM-1 and was shown to directly bind dsDNA via its Zα and Zβ DNA binding domains that are homologous to the nucleic acid binding domain adenosine deaminase acting on RNase1 (ADAR1) of an RNA-editing protein. Although DAI binds left-handed Z-form DNA with high affinity, it also binds B-form DNA and activates phosphorylation of IRF3 via TBK-1 serine/threonine kinase [56]. Inhibition of DAI using siRNA reduced activation of IFN-I production in response to human herpesvirus 1 (HSV-1), suggesting its potential role in detecting and mounting innate immune responses to DNA viruses.

Most recently, four groups independently reported the identification of a protein absent in melanoma 2 (AIM2) as a specific sensor of dsDNA in the cytoplasm [57–60]. AIM2 binds dsDNA via its C-terminal HIN-200 (hematopoietic interferon-inducible nuclear proteins with a 200–amino acid repeat) domain and this binding leads to oligomerization of the protein [61]. The N-terminal PYD domain of oligomerized AIM2 is capable of recruiting both ASC and caspase-1 inflammasome components and drives the activation of caspase-1 that then leads to IL-1β pre-protein processing and release of mature IL-1β. Knockdown of Aim2 abrogates caspase-1 activation in response to cytoplasmic dsDNA and the dsDNA vaccinia virus *in vitro*. However, AIM2 appears to not be normally present in the cytoplasm of uninfected cells and its expression depends on cell stimulation with IFN-I. This data suggests that, although AIM2 is critical for activation of inflammasome by dsDNA, it is unlikely to play a role in early recognition of viral infection. Instead, early stages of virus infection may activate IFN-I, which in turn leads to AIM2 expression that assists executing phases of innate immunity activation, such as processing and release of pre-synthesized IL-1β, if dsDNA is present in the cytoplasm.

## 3. Specifics of Innate Host Responses to Viral Gene Therapy Vectors

The existence of an array of specific receptors enabling detection of viruses within the cell is counterbalanced by numerous evasion strategies and mechanisms developed by virus pathogens. For instance, hepatitis C virus (HCV) encodes protein NS3/4A that cleaves IPS-1 and thus prevents IFN-I induction upon virus infection [62,63]. The packaging of viral genomic nucleic acids in association with virus-encoded DNA- or RNA-binding proteins may also represent a strategy to avoid detection of viral genomes in infected cells by TLRs, NLRs, or RLRs.

To achieve the goal of correcting human diseases, viral vectors for gene therapy must be able to safely and effectively deliver therapeutic genes to disease sites. However, the incorporation of therapeutic genes, along with all the necessary regulatory elements to guide tissue-specific transgene expression, often requires deletion of viral genes that are dispensable for vector production. Moreover, many viral vectors are based on attenuated forms of viruses that were specifically modified to reduce their virulence and prevent the re-emergence of their fully virulent forms. Most frequently such modifications of viruses are made at the expense of viral genes that counteract host innate and adaptive immunity. This makes viral vectors more prone to induce strong innate and/or adaptive immune responses. Another important consideration that may play a role in the induction of potent innate immune and inflammatory responses to viral vectors is that drastically higher doses of virus particles are delivered *in vivo* for therapeutic gene transfer, compared to doses that initiate natural virus infection. Indeed, a handful of wild type adenovirus particles are sufficient to induce a respiratory disease but in gene therapy trials adenovirus vectors may be delivered either locally or systemically at a single bolus dose of >10×10^12^ virus particles [1]. Clearly, the exposure of host cells to such a massive number of virus particles could initiate host responses that may be vastly distinct in magnitude and severity compared to those observed upon natural virus infection. One of the currently emerging concepts implies that due to the high vector doses that are needed to achieve efficient gene transfer and therapeutic gene expression, the activation of innate immunity to gene therapy vectors occurs due to the engagement of mechanisms that are naturally responsible for the detection of host cell damage or stress.

## 4. Innate Responses to Adenovirus Vectors

Adenovirus vectors (Ads) are the second most frequently used vectors in clinical trials in the US to treat numerous inborn and acquired human diseases, including cystic fibrosis and cancer [64]. Interest in Ad has recently increased considerably due to its potential as a vector for vaccination against life threatening infectious agents including anthrax [65,66]. Many of these potential applications will ultimately require intravenous Ad administration to achieve the desired therapeutic goals. Since a patient tragically died due to disseminated intravascular coagulation, systemic inflammation and multiple organ failure after Ad administration via the hepatic artery during a gene therapy trial in 1999 [3], major concerns have risen regarding the safety of systemic Ad injection. A decade later, the mechanistic aspects of and molecular pathways involved in triggering acute anti-Ad inflammatory response still remain poorly defined. Recent studies strongly suggest that the immediate innate immune response towards intravenously delivered Ads consists of several discrete components and include cytokine activation, pro-inflammatory Mϕ death, and influx of inflammatory leukocytes into affected sites.

### 4.1. Molecular mediators of early innate immune responses to Ad vectors

Even though natural infections with Ads are largely harmless in humans, an intravenous Ad vector administration for gene delivery purposes, especially at high doses, stimulates strong innate and adaptive immune responses and can be fatal for the host [1–3,67,68]. Upon systemic application of Ads in rodents, rhesus monkeys, and humans, a rapid liver-mediated vector removal from circulation was observed [68–74]. After intravenous delivery, Ads induce two phases of inflammatory gene expression in the liver. The first phase of acute inflammation occurs within 24 h of virus administration, and is entirely dependent on virus capsid interactions with the host cells [75]. The second phase begins 3–4 days after Ad administration and requires viral gene expression [76]. In animal models, intravenous Ad administration has been shown to induce transcription and release into the blood of a number of cytokines and chemokines, including IFN-I, IL-6, TNF-α, RANTES, IP-10, IL-8, MIP-1α, MIP-1β and MIP-2 [76–82]. Macrophages, including tissue residential macrophages (e.g., Kupffer cells in the liver), and dendritic cells throughout the body are considered to be the primary source of these cytokines and chemokines following their transduction with Ads [75]. Additionally, a rapid clearance of Ad from circulation by Kupffer cells may have a protective role against the dissemination of Ads to lymphoid organs, therefore reducing systemic inflammation. In several gene therapy clinical trials, serum levels of IL-6, IL-10 and IL-1 were elevated [83–86] after intravenous Ad administration at high doses (2×10^12^ – 6×10^13^ virus particles). The role of these cytokines in the initiation of an immediate innate immune response remains unclear. Histological evaluation of tissues, including lung, liver, and spleen, revealed areas of leukocyte and neutrophil infiltration as well as infarcts ([79,87], and our unpublished observation), indicating that most tissues in the body are involved in the inflammatory response to Ad after intravenous virus injection. It has been widely accepted that Ad-mediated liver damage plays a central role in the pathogenesis of acute systemic inflammation caused by intravenous Ad administration. To this end, it has been found that the activation of MIP-2 chemokine is at least partially responsible for neutrophil attraction to liver tissue, and that inactivation of MIP-2 with an anti-MIP-2 antibody ameliorates liver pathology after intravenous Ad administration [79]. To date, accumulating evidence suggests that Ad triggers highly complex multifaceted innate immune and inflammatory responses, which are reflected at a clinical level in cytokinemia, thrombocytopenia, complement activation, disseminated intravascular coagulation, and multiple organ failure due to (at least in part) collateral damage from infiltrating pro-inflammatory leukocytes. Despite considerable recent progress in defining early mediators of Ad-induced inflammation [26,52,88–90], the unifying mechanistic description of the sequential events that lead from early Ad-host cell interactions to clinical signs of Ad-triggered systemic toxicity remains illusive.

### 4.2. Molecular sensors of Ad infection

Using microarray technology, it was found that adenovirus infection rapidly dysregulates expression of up to 15% of all mRNA transcripts in mouse liver tissue or in human epithelial A549 cells [91,92]. These data prompted the search for intracellular sensor molecules that might be involved in recognition of adenovirus capsid components that can be collectively called molecular sensors of adenovirus infection.

From earlier studies it was established that the expression of genes involved in innate immune and inflammatory responses was significantly up-regulated shortly after Ad vector delivery, but prior to initiation of viral gene expression. In mouse and non-human primate models, Wilson and co-workers demonstrated activation of innate responses by transcriptionally-defective adenovirus particles [77,78]. Consistent with these findings, severe acute inflammatory response was also observed in a non-human primate model after the intravenous delivery of a helper-dependent Ad vector which lacked all viral genes [1]. These data directly demonstrate that the dose-dependent activation of innate immune and inflammatory responses are primarily mediated by adenovirus particle interaction with host cells and do not require viral gene expression.

Due to the central role of TLRs in detecting invading pathogens, considerable efforts were made to determine if this family of receptors is involved in the recognition of Ad infection. Recent data suggest that in response to Ad, plasmacytoid dendritic cells secrete type-I IFN in a TLR-9-dependent manner [93,94]. It is of interest that human cell lines expressing TLR9, permissive to infection by both coxsackievirus and adenovirus receptor (CAR)- and CD46-interacting Ad serotypes, showed a preferential activation of TLR9-mediated signaling by CD46-interacting serotypes [93]. These data are consistent with earlier findings that CD46-interacting Ads select an alternate intracellular trafficking pathway and reside in late endosomal compartments for longer times, compared to CAR-interacting Ad serotypes [95]. Because TLR9 expression is localized to late endosomal compartments, it appears that Ad DNA may activate TLR9 and induce type-I IFN expression. Using helper-dependent Ad vectors, Curello *et al.* found that the immediate innate immune response, assessed by plasma levels of IL-6 and IL-12, was partially attenuated in TLR9 knockout mice, when compared to the control group expressing wild type levels of TLR9 [96]. The involvement of TLR-mediated pathways in the induction of anti-Ad host responses was further analyzed in wild type and MyD88-knockout mice. These studies provided evidence that IFN-I is induced in response to Ad infection both in MyD88-dependent and -independent manner. For instance, plasmacytoid DCs produced IFNα/β in a TLR9 and MyD88-dependent manner, however, conventional DCs and macrophages initiated IFN-I production through a MyD88-independent mechanism, which likely involved an as yet unidentified cytosolic sensor of DNA [26]. In a very thorough study by Nociari *et al*., the authors provide further evidence that IFN-I expression in response to cell infection with Ad vectors occurs in a MyD88-independent manner. This data supports the idea that Ad DNA is likely detected by an unidentified sensor of nucleic acids in the cytoplasm [89]. This group provided compelling evidence that the Ad genomic dsDNA can induce phosphorylation of interferon regulatory factor IRF3, which is the key transcription factor in activating the antiviral IFN-I mediated signaling pathway. In a more recent paper, Fejer *et al*. demonstrated that plasmocytoid DCs are the principal source of type I IFN, which is activated 2–4 hours after intravenous Ad administration [88]. These authors also defined IRF7 as a critical mediator of Ad-induced IFN-I activation *in vivo*.

Recently, Muruve *et al*. reported that the NLRP3 inflammasome recognizes cytosolic microbial and host DNA and triggers the activation of host innate immune responses [52]. In addition to plasmid or bacterial DNA, the authors used Ad to demonstrate the involvement of NLRP3 inflammasome in cytosolic DNA sensing. Based on data obtained using *in vitro* systems 6 hours after virus infection, it was proposed that Ad is sensed by macrophages when virus particles reach for the cytosol and expose viral genomic DNA to the NLRP3 inflammasome sensor, which, via ASC, activates caspase-1 processing, that leads to IL-1β maturation and release. However, recent data from our laboratory strongly argues against the critical role of NLRP3 inflammasome in sensing Ad entry into cells *in vivo* and activating innate immune and inflammatory anti-Ad responses. Using a set of mice deficient for critical mediators of innate immunity and inflammation, we demonstrated that macrophage-derived IL-1α is the principal activator of the innate immune response to Ad *in vivo* [97]. Activation of IL-1α did not require MyD88-, TRIF-, or TRAF6-signaling, and occurred in mice deficient for IL-1β, IFN-IR, or inflammasome components caspase-1, ASC, and NLRP3. These findings strongly suggest that signaling pathways that were earlier implicated in the activation of an innate antiviral response and leading to IFN-I production [5] are not involved in triggering inflammatory and innate immune responses to Ad. Our studies also demonstrated that the IL-1α-mediated response critically depends on viral RGD motif-mediated binding to macrophage β_3_ integrins, which occurs prior to the internalization of the virus into the cell [98,99]. However, the magnitude of inflammatory response to Ad was greatly amplified by the virus-mediated endosome rupture. Because IL-1α is a key mediator of host inflammatory responses to dying cells [100], our studies strongly suggest that host cells trigger the activation of innate immunity to Ads via engaging cell damage or endosomal stress response mechanisms, rather than via sensing genomic Ad DNA within infected cells. However, in some specialized cell types like pDCs, sensing of Ad DNA by TLR9 may also contribute to the induction of innate anti-Ad responses [26]. Considering the major progress in our understanding of Ad-host interactions in recent years, it is conceivable that a comprehensive model of activation of innate immune and inflammatory responses by Ad *in vivo* will emerge in the near future.

## 5. Innate Responses to Adeno-Associated Virus Vectors

To date, numerous pre-clinical and clinical studies demonstrated that viral vectors based on adeno-associated virus (AAV) are relatively safe when delivered via an intravenous route and do not induce robust innate immune and inflammatory responses [101,102]. However, the host innate immune system plays a central role in shaping the outcome of a gene delivery using AAV-based vector systems [103]. The earlier studies clearly demonstrated that when injected intravenously into mice at high doses, AAV induces transcriptional activation of inflammatory cytokine and chemokine genes, including TNF-α, RANTES, MIP-1β, MIP-2, MCP-1, and IP-10. Moreover, the absolute amounts of these cytokines and chemokines in the liver 1 hour after virus injection were comparable for AAV and Ads [104]. Although the expression of these genes greatly receded by 6 hours after AAV (but not Ad) administration, this data indicates that the AAV cell entry process and/or early interactions with host cells triggers the activation of signaling pathways that initiate the unraveling of a stereotypic pro-inflammatory host response. The same study further showed that Kupffer cells were primarily responsible for the activation of these pro-inflammatory genes in the liver after AAV administration [104].

Because adaptive immunity represents a principal barrier for successful gene transfer using AAV-based vectors [103], much effort was made to understand the AAV interaction with professional antigen-presenting cells, specifically DCs. In a most recent and very detailed study by Zhu *et al*., the authors found that AAV is recognized by pDCs via TLR9-MyD88-dependent pathway [105]. As noted above, in this specialized cell type, TLR-MyD88 pathway is coupled to IRF7 activation that leads to IFN-I production. Zhu *et al*. further demonstrated that AAV-mediated induction of IFN-I in pDCs is completely dependent on TLR9 and MyD88 *in vitro*. Importantly, they further demonstrated that TLR9-MyD88 pathway was critical for the activation of CD8+ T cell responses to both the transgene product and AAV capsid *in vivo*, leading to the generation of both transgene product-specific and AAV capsid-specific neutralizing antibodies [105]. Collectively, these data are fundamental for our understanding of the AAV-based vector recognition by the host cells and for the development of approaches to modulate the innate immune system to reduce vector recognition and improve therapeutic transgene expression in the target cells.

## 6. Conclusions

The wealth of new data on molecular sensors of viral infection that emerged during the last two years creates an opportunity for the development of basic paradigms of virus infection recognition in mammalian cells. One of the fundamental principles of virus-host discrimination by the innate immune system appears to rely on recognition of virus-associated nucleic acids by the specialized families of receptors TLRs, RLRs, and NLRs. While the TLR receptors function at the cell surface and within the cellular endosomal compartments, RLR and NLRs function within the cellular cytoplasm. Although the engagement of TLRs and RLRs triggers the activation of signal transduction pathways leading to the inflammatory cytokine and chemokine gene expression, NLRs are critical for the executive stages of innate immunity activation and process inflammatory caspases and inflammasome-dependent cytokines.

The activation of innate immune responses to viral vectors also occurs via the engagement of TLRs and NLRs. However, in addition to direct detection of their genomic nucleic acids, host cells may recognize viral vectors though an indirect sensing of cell damage or stress that occurs due to a high vector dose delivery frequently employed in gene transfer protocols. Importantly, it was found that in response to viral vectors, different cell types activate distinct signaling pathways that ultimately shape the multifaceted innate immune and inflammatory responses observed in pre-clinical studies and clinical gene therapy trials. Future studies should focus on improving our understanding of cell type-specific pathways and mediators of inflammation in response to the viral vector administration *in vivo*. This information will be critical for the development of new approaches to selectively modulate these responses and ultimately to improve the safety of viral vector-mediated gene transfer for human gene therapy application.
